# Glycine-rich RNA binding protein of *Oryza sativa *inhibits growth of M15 *E. coli *cells

**DOI:** 10.1186/1756-0500-4-18

**Published:** 2011-01-26

**Authors:** Upasana Singh, Debadutta Deb, Amanjot Singh, Anil Grover

**Affiliations:** 1 Department of Plant Molecular Biology, University of Delhi South Campus, New Delhi, India

## Abstract

**Background:**

Plant glycine-rich RNA binding proteins have been implicated to have roles in diverse abiotic stresses.

**Findings:**

*E. coli *M15 cells transformed with full-length rice glycine-rich RNA binding protein4 (OsGR-RBP4), truncated rice glycine-rich RNA binding protein4 (OsGR-RBP4ΔC) and rice FK506 binding protein (OsFKBP20) were analyzed for growth profiles using both broth and solid media. Expression of OsGR-RBP4 and OsGR-RBP4ΔC proteins caused specific, inhibitory effect on growth of recombinant M15 *E. coli *cells. The bacterial inhibition was shown to be time and incubation temperature dependent. Removal of the inducer, IPTG, resulted in re-growth of the cells, indicating that effect of the foreign proteins was of reversible nature. Although noted at different levels of dilution factors, addition of purified Os-GR-RBP4 and OsGR-RBP4ΔC showed a similar inhibitory effect as seen with expression inside the bacterial cells.

**Conclusions:**

Expression of eukaryotic, stress-associated OsGR-RBP4 protein in prokaryotic *E. coli *M15 cells proves injurious to the growth of the bacterial cells. *E. coli *genome does not appear to encode for any protein that has significant homology to OsGR-RBP4 protein. Therefore, the mechanism of inhibition appears to be due to some illegitimate interactions of the OsGR-RBP4 with possibly the RNA species of the trans-host bacterial cells. The detailed mechanism underlying this inhibition remains to be worked out.

## Findings Background

RNA binding proteins (RBPs) are characterized by two important structural features: (1) presence of one or more RNA recognition motif [RRM: also known as RNA binding domain (RBD) or ribonucleotide protein domain (RNPs)] at the N-terminus and (2) a variety of auxillary motifs at the C-terminus such as glycine rich (GR) region, arginine rich region, acidic SR-repeats and RD-repeats [1]. In general, RRM is shown to be composed of two RNA binding consensus sequences namely RNPI [a highly-conserved octameric peptide stretch RGFGFVTF; (K/R)G(F/Y)(G/A)FVX(F/Y)] and RNPII [a relatively less-conserved hexameric peptide stretch CFVGGL; (C/I)(F/Y)(V/I)(G/K)(G/N)L]. Genome sequencing projects have revealed that RRM containing proteins are abundantly present in varied life forms including viruses, prokaryotes as well as eukaryotes. Proteins that contain RRMs at the N-terminus and GR domain (GD) at the C-terminus are referred to as glycine rich- RNA binding proteins (GR-RBPs). GD in general consists of an RGG and three GGYGG boxes (two complete; one incomplete). GR-RBPs have been identified in a variety of monocotyledon and dicotyledon plants including bean, *Arabidopsis*, tobacco, carrot, maize and rice [2-4]. These proteins have largely been implicated in cell functions linked to metabolism of mRNA molecules. These include processing, transport, localization, translation and stability of mRNAs. Strikingly, transcripts of GR-RBPs are noted to be up-regulated in response to number of stress stimuli including cold, water stress, high salinity, UV-radiations and heavy metals [3]. The expression of GR-RBPs has also been known to show diverse response to wounding, flooding, hormone and infection by pathogens [3]. *At*GR-RBP designated as RZ-1a has been proposed to play a role in the enhancement of freezing tolerance of *Arabidopsis *plants [5]. Kim *et al. *[6] produced transgenic *Arabidopsis *plants over-expressing RZ-1a using 35S-promoter. This work showed that RZ-1a has a negative impact on seed germination and seeding growth of transgenic *Arabidopsis *plants under salt or dehydration stress conditions. Kim *et al. *[6] further reported that GR-RBP2, in *Arabidopsis *has a positive impact on seed germination and seedling growth of plants under cold stress conditions. Kwak *et al. *[7] have reported that GR-RBP4 negatively affects seeds germination and seedling growth of *Arabidopsis *plants under salt and dehydration stress conditions. Recently, it has been shown that *Arabidopsis *GR-RBP7 has RNA chaperone activity during the process of cold adaptation in *E. coli *[6].

Agarwal [8] isolated a GR-RBP cDNA from rice heat shock cDNA library constructed using mRNA of heat shocked rice seedlings. Osgr-rbp cDNA isolated in this study was designated as OsGR-RBP4 [4]. Osgr-rbp4 transcript was seen to be up-regulated in response to heat stress in rice. Sahi *et al. *[4] further reported that recombinant yeast cells over-expressing full-length Osgr-rbp4 showed increased basal high temperature tolerance as compared to the wild type cells or cells transformed with vector backbone. There are indications that heat stress increases the stability of specific mRNAs [9]. Noon *et al. *[10] reported that post-transcriptional modifications in tRNAs and rRNAs are especially abundant in thermophilic organisms and these modifications appear to play a functional role in structural stabilization of RNA at elevated temperatures. Sahi *et al. *[4] argued that OsGR-RBP4 may have provided increased high temperature protection to yeast cells through its binding to mRNA molecules. Agarwal [8] as well as Sahi *et al. *[4] from our laboratory have earlier observed that bacterial cells transformed with full-length Osgr-rbp4 are slower in growth under culturing conditions. We provide detailed analysis of the expression of Osgr-rbp4 in M15 *E. coli *cells, in this study.

## Methods

### Construction of recombinant *E. coli *strains

*E. coli *strain M15 [pREP4] (Qiagen) which permits high levels of protein expression was used in this study. M15 contains a low-copy pREP4 plasmid which confers kanamycin resistance and constitutively expresses the *lac *repressor protein encoded by *lac I *gene. *E. coli *M15 strain does not contains a chromosomal copy of *lac I *and therefore, pREP4 must be maintained by selection for kanamycin resistance. The cells were cultured in LB broth (Luria-Bertani medium, USB Corporation, USA) with kanamycin (kan, 50 μg mL^-1^) for antibiotic selection. pQE30 (Qiagen) was used for expression of OsGR-RBP4 protein as well as OsFKBP20 protein which was used as a positive control. pQE30 contains β-lactamase gene (bla) which confers resistance for ampicillin. Carbenicillin is a semi-synthetic analog of ampicillin and can be used in place of ampicillin. We used carbenicillin in place of ampicillin because of its stability. PCR was performed to confirm the presence of the foreign insert into *E. coli *M15 cells and subsequently, sequencing was performed to confirm the fidelity of the cloned products. In order to analyze the expression of foreign insert into the *E. coli *M15 cells, RNA was isolated and RT-PCR was performed. For RNA isolation, M15 cells containing OsGR-RBP4, OsGR-RBP4ΔC, OsFKBP20/, and pQE30 constructs were grown overnight at 37°C in LB broth with carbenicillin and kanamycin. Secondary cultures were put up after ~16 h of incubation of primary cultures by measuring the O.D. of primary culture at 600 nm using Hitachi U-2810 spectrophotometer and diluting the cultures to an O.D. of 0.5. After 3 h of initiation of secondary culturing, cultures were divided into two parts: one part was induced with 1 mM IPTG while the other served as a control. The cultures were pelleted at 6000 rpm for 3 min at 4°C and RNA was isolated as per standard procedures. The RNA so obtained was used to make cDNA. cDNAs were synthesized for semi-quantitative RT-PCR from 2 μg of total RNA using MMLV reverse transcriptase (Promega, USA). The following primers were used for amplifying the genes; for both OsGR-RBP4 and OsGR-RBP4ΔC: Forward 5' cgcctgggccaccgacgac3' and reverse 5'ggagcggcgcgactgggcct3'; OsFKBP20:Forward 5'ggaattcatggcagaggttgcagattt3' and reverse 5'ggggtaccttatttctcttcctccttg3'.

To confirm the protein expression from OsGR-RBP4M15, OsGR-RBP4ΔCM15, OsFKBP20M15, and pQE30/M15 cells, secondary cultures were put up from the primary cultures as described earlier. After 3 h of initiation of secondary culturing, cultures were divided into two parts: one part was induced with 1 mM IPTG while the other served as a control. The two sets were pelleted, resuspended in Laemmli buffer and loaded on SDS-PAGE gel (12% resolving, 3.9% stacking). The gel was stained with Coomassie brilliant blue R (CBB-R).

### Growth analysis of transformed *E. coli *cells

*E. coli *M15 cells containing OsGR-RBP4, OsGR-RBP4ΔC, OsFKBP20, and pQE30 constructs were grown overnight at 37°C in LB broth containing kanamycin and carbenicillin. After approximately 16 h of incubation of primary culture, secondary culture was put up and induced with 1 mM IPTG as mentioned above. After every 30 min, O.D. (at 600 nm) of the culture was determined and growth curve was plotted. For the plate assays of *E. coli *cells, secondary cultures for *E. coli *M15 cells containing OsGR-RBP4, OsGR-RBP4ΔC, OsFKBP20 and pQE30 constructs were put up after approximately 16 h of incubation of primary culture and induced with IPTG as mentioned earlier. After every 30 min, 10 μl culture was diluted in 90 μl of water to make a dilution of 10^-1 ^which was further diluted 2 times to obtain 10^-2 ^and 10^-3 ^dilutions. 5 μl of these dilutions were spotted on LB agar plates with carbenicillin and kanamycin as selection and were allowed to be absorbed by the plates. The plates were made for -IPTG, +IPTG 30 min, +IPTG 60 min, +IPTG 90 min and +IPTG 120 min treatments.

### Plate assay of *E. coli *cells exogenously provided with OsGR-RBP4, OsGR-RBP4ΔC and OsFKBP20 proteins

Secondary cultures for *E. coli *M15 cells were initiated after approximately 16 h of incubation of primary culture and induced with IPTG as mentioned earlier. Purified OsGR-RBP4, OsGR-RBP4ΔC and OsFKBP20 proteins as of above were quantified using spectrophotometer at 595 nm and equal amount of proteins (120 μg) were added to a 3 mL culture containing equal number of M15 cells (using equal O.D. values at A_600_). Cultures were kept on continuous shaking at 37°C for approximately 8 h. After 8 h, 10 μl of each culture were diluted in 90 μl of water to make a dilution of 10^-1 ^which was further diluted 11 times to obtain 10^-2 ^to 10^-12 ^dilutions. 5 μl of these dilutions (10^-5 ^to 10^-12^) were spotted on LB agar plates with kanamycin as selection and plates were incubated at 37°C.

### Analysis of the sequences and homology search

Protein sequences were analysed using DNA analysis software DNASTAR. Predicted protein sequences were determined using DNASTAR software and deduced amino acid sequences were searched for their homology with the previously existing sequences in the NCBI database using the BlastP program [11].

## Results

### Constructs, transformation and expression analysis

**Figure 1:**
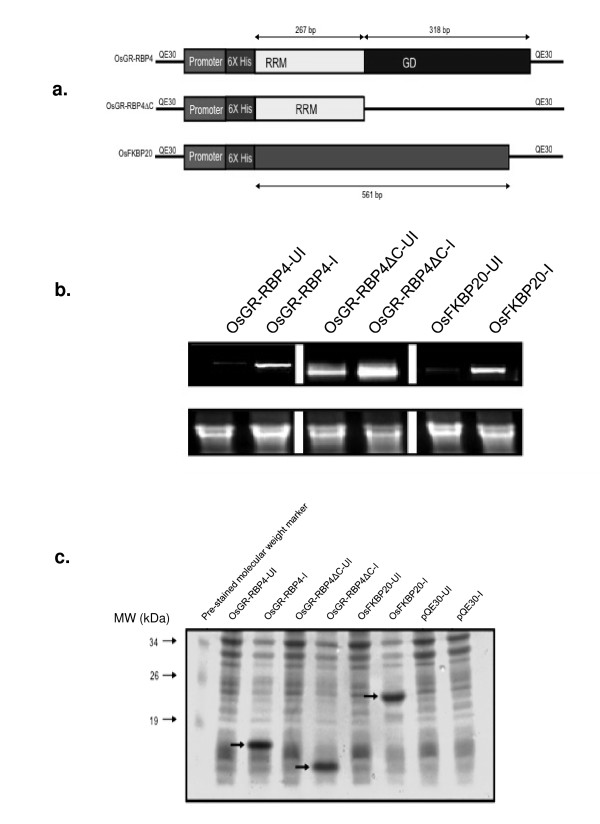
**Design of constructs, confirmation of transcription and translation of rice proteins in *E. coli***. **a**. Linear maps of the recombinant constructs employed in this study. **b**. RT-PCR analysis of IPTG un-induced and induced samples of transformed *E. coli *M15 cells. Upper panel shows RT-PCR depicting expression of the respective genes. Lower panel shows EtBr stained gel depicting comparable staining of the ribosomal RNA species, 2 μg of total RNA was loaded in each lane. **c**. Expression of the foreign proteins in the transformed *E. coli *cells. Total protein from the IPTG un-induced and induced cells were separately analyzed. Induced proteins are indicated by arrows. Pre-stained markers are shown on the left side of the panel. UI and I refer to IPTG un-induced and induced samples, respectively.

This study aimed at the analysis of the OsGR-RBP4 expression in *E. coli *M15 cells. To accomplish this, OsGR-RBP4 cloned in pQE30 vector was used. We also used OsGR-RBP4ΔC (truncated version of the Osgr-rbp4 with intact RRM but lacking the glycine rich domain sequences) cloned in pQE30. In addition, we used OsFKBP20 cDNA cloned in pQE30 as control (Figure 1a). As a plasmid control, pQE30 was also transformed in *E. coli *M15 cells. Four types of recombinant *E. coli *cells thus analyzed were as per the following constitution: OsGR-RBP4 in M15 cells (OsGR-RBP4/M15), OsGR-RBP4ΔC/M15, OsFKBP20/M15 and pQE30/M15. Selection of the transformed bacterial cells on antibiotic provided first line of evidence that the cells were transformed. Further, expression of the foreign inserts in the *E. coli *M15 host cells were examined at the transcript level. RNAs isolated from OsGR-RBP4/M15, OsGR-RBP4ΔC/M15 and OsFKBP20/M15 cells were used for cDNA preparation. cDNAs were used for RT-PCR reactions and the products of these reactions were analyzed on 1% agarose gel. As shown in Figure 1b, clear expression was noted for OsGR-RBP4, OsGR-RBP4ΔC and OsFKBP20 RNAs in RT-PCR reaction from the IPTG-induced cells. It is noteworthy that low level expression was also apparent for the IPTG un-induced samples of various transformants (particularly for the OsGR-RBP4ΔC/pQE30 lane). Subsequently, expression of the foreign protein was examined in the *E. coli *M15 host cells. Total proteins isolated from OsGR-RBP4/M15, OsGR-RBP4ΔC/M15 and OsFKBP20/M15 cells were loaded on 12% SDS-gel. As shown in Figure 1c, the expressed foreign proteins were clearly noted in the IPTG induced samples (shown by arrows). No such protein was detected in *E. coli *M15 cells transformed with pQE30 plasmid backbone.

### Growth analysis of the *E. coli *M15 cells

**Figure 2:**
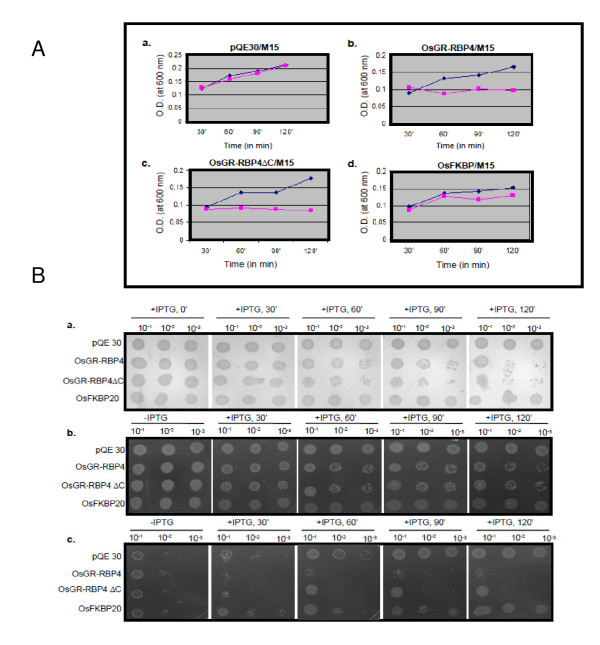
**Dynamics of *E. coli *before and after induction with IPTG**. **A**. Growth profile of *E. coli *M15 cells. Panels **a, b, c **and **d **show growth profiles of pQE30/M15, OsGR-RBP4/M15, OsGR-RBP4ΔC/M15 andOsFKBP0/M15 *E. coli *cell types, respectively. Purple line denotes un-induced and blue line denotes induced *E. coli *M15 cells. **B. a**. Plate culture assay of OsGR-RBP4/M15, OsGR- RBP4PΔC/M15, OsFKBP20/M15 and pQE30/M15 *E. coli *cells (incubation at 37°C). Cells were harvested at various time points after IPTG induction as shown. Serial dilutions were made and equal number of cells was spotted on LB agar plates with antibiotic selection. Plates were photographed after 16 h of incubation. +IPTG refers to induction by IPTG for the time periods indicated at the top. **b**. Plate assay of OsGR-RBP4/M15, OsGR-RBP4PΔC/M15, OsFKBP20/M15 and pQE30/M15 *E. coli *cells (incubation temperature of the plates = 20°C). Cells were harvested at various time points after IPTG induction as shown. **c**. Plate assay of OsGR-RBP4/M15, OsGR-RBP4PΔC/M15, OsFKBP20/M15 and pQE30/M15 *E. coli *cells, (incubation temperature of the plates = 45°C).

Next, growth profiles of OsGR-RBP4/M15, OsGR-RBP4ΔC/M15, OsFKBP20/M15 and pQE30/M15 cells were monitored spectrophotometrically both for the IPTG un-induced and the induced cultures. The overnight grown primary cultures were used to set the secondary cultures beginning with 0.5 O.D. O.D. values were subsequently recorded after every 30 min interval. Growth curves were then plotted for the O.D. values against time intervals. From Figure 2A, it is clear that the growth rates of all four cells types (OsGR-RBP4/M15, OsGR-RBP4ΔC/M15, OsFKBP20/M15 and pQE30/M15) were nearly comparable under IPTG un-induced conditions (shown by blue line color). Strikingly, OsGR-RBP4/M15 and OsGR-RBP4ΔC/M15 cells types after IPTG induction failed to grow at all on secondary culturing (shown by pink line color). There was no effect of IPTG addition to pQE30/M15 cell type. While OsFKBP20/M15 cells did grow upon IPTG induction, its growth was slightly less than pQE30/M15 cell type.

To reaffirm the above observations, 5 μl cells from the IPTG un-induced and induced culture were spotted after serial dilutions (10^-1^-10^-3^) on LB agar plates with antibiotic selection and allowed to be absorbed by the medium. Similar to the observations made with broth culture, plate assay showed that induction with IPTG has detrimental effect on the viability of OsGR-RBP4/M15 and OsGR-RBP4PΔC/M15 cells. These cells upon culturing on plates showed reduced viability. On the other hand, OsFKBP20/M15 and pQE30/M15 cell types showed no such inhibitory effects (Figure 2B.a).

**Figure 3:**
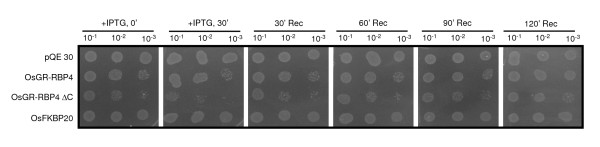
**Assays for *E. coli *viability**. Plate assay of OsGR-RBP4/M15, OsGR-RBP4PΔC/M15, OsFKBP20/M15 and pQE30/M15 *E. coli *cells after the washing off the IPTG. Other details same as in Figure 2B.

Next we tested the effect of temperature on bacterial growth. Secondary culture (0.5 O.D. of the overnight grown primary culture used to set the secondary culture) was set up for all the four transformed *E. coli *M15 cell types. After 3 h, total culture volume was divided into two parts: one part was induced by IPTG and other was left un-induced. After every 30 min interval, cells from the un-induced and induced cultures were serially diluted and spotted on LB agar plates with carbenicillin and kanamycin. Three sets of plates were prepared and one set of each of these was incubated for 16 h at 20°C, 37°C and 45°C in separate incubators. At 20°C (Figure 2B. b) and 37°C (Figure 2B. a), all the four cells types showed comparable growth profile in the sets made without induction by IPTG (Figure 2B. b). In sets prepared with IPTG induced cell types, OsGR-RBP4/M15 and OsGR-RBP4ΔC/M15 cells showed lower growth profiles than OsFKBP20/M15 and pQE30/M15 cell types (Figure 2B. c). At both the temperatures of plate incubation, this effect was more pronounced after at least 60 min of IPTG induction. At 45°C (Figure 2B.c), increased lethality to OsGR-RBP4/M15 and OsGR-RBP4ΔC/M15 cells as against OsFKBP20/M15 and pQE30/M15 cells was apparent even at 30 min of IPTG induction. In this stress regime, it was further noted that OsFKBP20M15 and pQE30/M15 cells were able to grow gradually with time following induction by IPTG. However, OsGR-RBP4/M15 and OsGR-RBP4ΔC/M15 cells failed to overcome the lethality effects following IPTG induction (Figure 3).

In subsequent experiment, IPTG was washed off from the cultures of all four cell types after inducing it with IPTG for 30 min. The recovered cell cultures were serially diluted and spotted on LB agar plates with antibiotic selection (plates incubated at 37°C for 16 h). The lethality noted for OsGR-RBP4/M15, OsGR-RBP4PΔC/M15 cells was alleviated to a significant extent upon IPTG washing (Figure 3).

**Figure 4:**
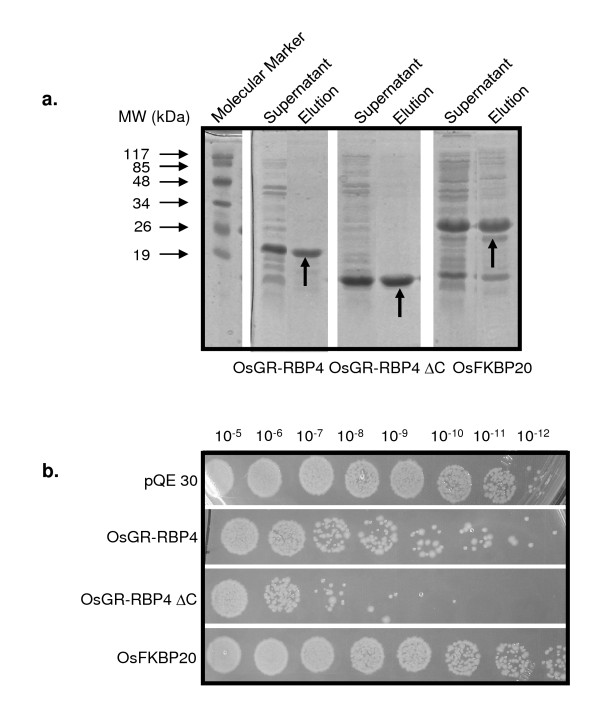
**Affect of affinity purified proteins on bacterial growth**. **a**. Purification of OsGR-RBP4, OsGR-RBP4PΔC and OsFKBP20 proteins by affinity column chromatography. Arrows indicate the presence of partially purified proteins in respective panels. Pre-stained markers are shown on the extreme left panel. **b**. Effect of purified OsGR-RBP4, OsGR-RBP4PΔC and OsFKBP20 proteins on the survival of *E. coli *M15 cells under *in vitro *conditions. Photograph shows serial dilutions made after 8 h and spotted on LB agar plates with antibiotic selection.

Further, growth profiles of *E. coli *M15 cells were monitored in response to addition of purified OsGR-RBP4, OsGR-RBP4PΔC and OsFKBP20 proteins. The respective proteins were partially purified from the corresponding cultures using affinity column chromatography (respective proteins shown by arrows in Figure 4a). Equal amounts of proteins (120 μg) were added directly into the *E. coli *M15 cultures. It may be noted here that because of the variations in the molecular weights of the purified OsGR-RBP4, OsGR-RBP4PΔC and OsFKBP20 proteins, the number of individual protein molecules in 120 μg of protein may vary. After ~8 h of growth, cultures were spotted on LB agar plates with selection (kan). Exogenous addition of the OsGR-RBP4 and OsGR-RBP4PΔC proteins showed a distinct toxic effect on the growth of *E. coli *M15 cells. Out of these two, OsGR-RBP4PΔC appeared more toxic to cells than OsGR-RBP4. This again may be possible because of higher ability of OsGR-RBP4PΔC protein to enter into *E. *coli owing to its lower molecular weight. On the other hand, exogenous addition of OsFKBP20 protein didn't result in growth inhibition of bacterial cells (Figure 4b).

Next, we were interested to see if there is any protein in *E. coli *genome homologous to OsGR-RBP4. OsGR-RBP4 sequence was searched for their homology with *E. coli *in the NCBI database using BlastP program. We didn't observe any significant homology of OsGR-RBP4 to any *E. coli *protein in this analysis.

## Discussion

*E. coli *is a host of choice for expression of eukaryotic proteins. Large number of proteins from diverse eukaryotes has been expressed in *E. coli*. On the other hand, there are instances in which expression of selective eukaryotic proteins in *E. coli *have been shown to exert toxic effects on the growth of host *E. coli *cells. In such cases, the expression yields of the proteins are dramatically diminished or sometimes abolished. The striking examples of this class include (1) aspartic protease from human immunodeficiency virus type I [12], (2) recombinant feline TNF [13], (3) malarial oritidine-5'-monophosphatedecarboxylase [14] and (4) phage HK 022 nun protein [15]. There are indications that such genes interfere with the physiology of *E. coli*. A few plant proteins have also been reported to have deleterious effect on the growth of the trans-host. Habuka *et al. *[16] observed that overexpression of *Mirabilis *antiviral protein (MAP) weakly-inhibits protein synthesis in *E. coli *which further results in a decrease in the growth of the bacterial cells. Girbes *et al. *[17] found that Ebulin1(a type 2 ribosome inactivating protein) from *Sambucus ebulus *strongly inhibits protein synthesis in rabbit reticulocyte lysate, rat brain and rat liver cell free systems but does not affect plant or bacterial protein synthesis *in vitro*. In this study, we noticed that expression of OsGR-RBP4 in *E. coli *M15 cells adversely affects the growth of the host cells. This effect was specifically caused by OsGR-RBP4 protein as (1) growth inhibition of the host *E. coli *cells was apparent only when cells were induced by IPTG and (2) there was no inhibition in growth of *E. coli *cells when either OsFKBP20 or backbone vector sequence pQE30 were expressed. OsGR-RBP4 protein consists of RBD at N-terminus and GD at C-terminus. We removed GD sequence from the full-length OsGR-RBP4 sequence to construct a GD-deleted strain (i.e. OsGR-RBP4ΔC). When OsGR-RBP4ΔC was expressed in *E. coli *cells, a similar decrease in the growth was observed (Figures 2 and 3), suggesting that the deleterious effect of OsGR-RBP4 in *E. coli *cells is possibly contributed by the RBD. It appears that the inhibitory effect of OsGR-RBP4 and OsGR-RBP4ΔC proteins is a time-dependent process i.e. there was not much difference in the growth of the OsGR-RBP4/M15 and OsGR-RBP4ΔC/M15 cells up to 60 min of IPTG addition but beyond 60 min time interval of IPTG addition there was a significant percent decrease in the growth of the above cell types. This may be related to the expression/accumulation of the requisite proteins. We have used proteins with an N-terminal 6-histidine tag. Although it has been observed that it is important to maintain native protein structure for protein folding/structural studies, we did not come across any report mentioning proteotoxicity resulting because of histidine tag.

On altering the incubation temperature of the plates, it was further noted that while plates incubated at 20°C and 37°C showed drastic effects with 60 min of IPTG treatment, plates incubated at 45°C showed growth inhibitory effects even with 30 min of IPTG treatment. It is possible that this inhibitory effect has a correlation with the metabolic state of the cells: cells with higher metabolic status/ growth rates at increased temperature appear more vulnerable to inhibitory effects.

The principal activity of the OsGR-RBP4 appears to be its binding to RNA species. The toxic effects of expression of OsGR-RBP4 (as well as OsGR-RBP4ΔC) may be due to its binding to some vital bacterial transcripts. As we didn't find any natural homolog of the OsGR-RBP4 protein in *E. coli*, it is possible that expression of this eukaryotic protein may be involved in some illegitimate binding with bacterial transcripts. It also needs to be considered that this binding may be of flux nature because when IPTG is washed off, re-growth of the OsGR-RBP4M15 and OsGR-RBP4ΔC/M15 cells was noticed in this study. It is generally considered that higher accumulation of the foreign proteins may lead to protein toxicity due to higher demand for energy and molecular machinery for the degradation. We also noted that OsFKBP20 expression resulted in lesser growth of the host cells compared to pQE30/M15 cells. However, the increased arrest of growth noted in OsGR-RBP4/M15 and OsGR-RBP4ΔC/pQE30/M15 cells as compared to OsFKBP20/M15 cells suggests that expression of GR-RBP causes specific interference in RNA metabolism and thus growth of bacterial cells expressing this protein is drastically affected.

This study was undertaken with only the *E. coli *M15 cells. Testing of the other strains of *E. coli *as well as other gram-negative or gram-positive bacteria would be interesting. If OsGR-RBP4 turns out to be broad spectrum bactericidal protein, it may have applied values. It would be worth to assess the toxicity of rice OsGR-RBP4 protein on disease causing bacterial strains. In future we plan to do this kind of analysis using *Nicotiana tabacum *transgenics raised in our laboratory over-expressing OsGR-RBP4 protein

## Competing interests

The authors declare that they have no competing interests.

## Authors' contributions

US and DD carried out the experiments and took part in the discussion. AG and AS designed the experiments and drafted the manuscript alongwith US. All the authors read and approved the final manuscript.

## References

[B1] Alba MM Pages M Plant proteins containing the RNA-recognition motif Trends in Plant Science 1998 3 15 21 10.1016/S1360-1385(97)01151-5

[B2] Gomez J Sanchez-Martinez D Stiefel V Rigau J Puigdomenech P Pages M A gene induced by the plant hormone abscisic acid in response to water stress encodes a glycine-rich protein Nature 1998 334 262 264 10.1038/334262a0 2969461

[B3] Sachetto-Martins G Franco LO de Oliveira DE Plant glycine-rich proteins: a family or just proteins with a common motif? Biochim Biophys Acta 2000 1492 1 14 10858526 10.1016/s0167-4781(00)00064-6

[B4] Sahi C Agarwal M Singh A Grover A Molecular characterization of a novel isoform of rice (*Oryza Sativa *L.) glycine-rich RNA binding protein and evidence for its involvement in high temperature stress response Plant Science 2007 173 144 155 10.1016/j.plantsci.2007.04.010

[B5] Kim YO Kim JS Kang H Cold-inducible zinc finger-containing glycine-rich RNA-binding protein contributes to the enhancement of freezing tolerance in *Arabidopsis thaliana* Plant J 2005 42 890 900 10.1111/j.1365-313X.2005.02420.x 15941401

[B6] Kim JS Park SJ Kwak KJ Kim YO Kim JY Song J Jang B Jung CH Kang H Cold shock domain proteins and glycine-rich RNA-binding proteins from *Arabidopsis thaliana *can promote the cold adaptation process in *Escherichia coli* Nucleic Acids Res 2007 35 506 516 10.1093/nar/gkl1076 17169986 PMC1802614

[B7] Kwak KJ Kim YO Kang H Characterization of transgenic *Arabidopsis *plants overexpressing GR-RBP4 under high salinity, dehydration, or cold stress J Exp Bot 2005 56 3007 3016 10.1093/jxb/eri298 16207746

[B8] Agarwal M Molecular characterization of high and low molecular weight heat shock genes/proteins from rice (*Oryza sativa *L.) 2002 Ph.D. thesis submitted to University of Delhi

[B9] Laroia G Cuesta R Brewer G Schneider RJ Control of mRNA decay by heat shock-ubiquitin-proteasome pathway Science 1999 284 499 502 10.1126/science.284.5413.499 10205060

[B10] Noon KR Bruenger E McCloskey JA Posttranscriptional modifications in 16S and 23S rRNAs of the archaeal hyperthermophile *Sulfolobus solfataricus* J Bacteriol 1998 180 2883 2888 9603876 10.1128/jb.180.11.2883-2888.1998 PMC107253

[B11] Altschul SF Madden TL Scheffer AA Zhang Z Moller W Lipman DJ Gapped BLAST and PSI-BLAST: a new generation of protein database search programs Nucl Acids Res 1997 25 3389 3402 10.1093/nar/25.17.3389 9254694 PMC146917

[B12] Korant BD Rizzo CJ An *E. coli *expression system which detoxifies the HIV protease Biomed Biochim Acta 1991 50 643 646 1801736

[B13] Otto CM Niagro F Su X Rawlings CA Expression of recombinant feline tumor necrosis factor is toxic to *Escherichia coli* Clin Diagn Lab Immunol 1995 2 740 746 8574840 10.1128/cdli.2.6.740-746.1995 PMC170231

[B14] Cinquin O Christopherson RI Menz RI A hybrid plasmid for expression of toxic malarial proteins in *Escherichia coli* Mol Biochem Parasitol 2001 117 245 247 10.1016/S0166-6851(01)00354-1 11606237

[B15] Uc-Mass A Khodursky A Brown L Gottesman ME Overexpression of phage HK022 Nun protein is toxic for *Escherichia coli* J Mol Biol 2008 380 812 819 10.1016/j.jmb.2008.05.030 18571198 PMC2597491

[B16] Habuka N Murakami Y Noma M Kudo T Horikoshi K Amino acid sequence of *Mirabilis *antiviral protein, total synthesis of its gene and expression in *Escherichia coli* J Biol Chem 1989 264 6629 6637 2708328

[B17] Girbes T Citores L Iglesias R Ferreras JM Munoz R Rojo MA Arias FJ Garcia JR Mendez E Calonge M Ebulin 1, a nontoxic novel type 2 ribosome-inactivating protein from *Sambucus ebulus *L. leaves J Biol Chem 1993 268 18195 18199 8349695

